# Supplementary material for: ADCC against MICA/B Is Mediated against Differentiated Oral and Pancreatic and Not Stem-Like/Poorly Differentiated Tumors by the NK Cells; Loss in Cancer Patients due to Down-Modulation of CD16 Receptor
Source: Cancers (Basel). 2021 Jan 11;13(2):239. doi: 10.3390/cancers13020239 (PMC7826810; doi:10.3390/cancers13020239)
Supplement: Supplementary file 1 [file cancers-13-00239-s001.pdf]

Supplementary Data

ADCC against MICA/B is mediated against differentiated oral and pancreatic and not stem-like/poorly differentiated tumors by the NK cells; Loss in cancer patients due to downmodulation of CD16 receptor

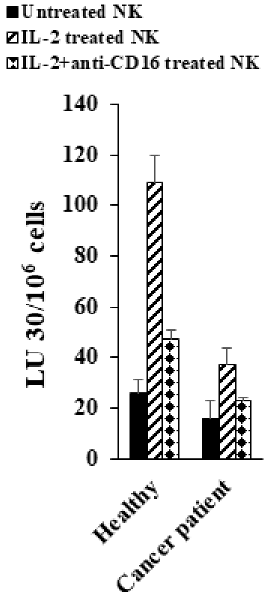

Figure S1. Cancer patients' NK cells exhibit lower cytotoxicity against K562 tumors. Purified NK cells (1 × 10^6 cells/ml) from healthy individuals and cancer patients were left untreated, treated with IL-2 (1000 U/ml), or treated with a combination of IL-2 (1000 U/ml) and anti-CD16 mAb (3 μg/ml) for 18 hours and were added to ^{51}Cr-labeled K562 at various effector-to-target ratios. NK cell-mediated cytotoxicity was measured using a standard 4-hour ^{51}Cr release assay against K562. The lytic units (LU) 30/10^6 cells were determined using the inverse number of NK cells required to lyse 30% of target cells × 100.

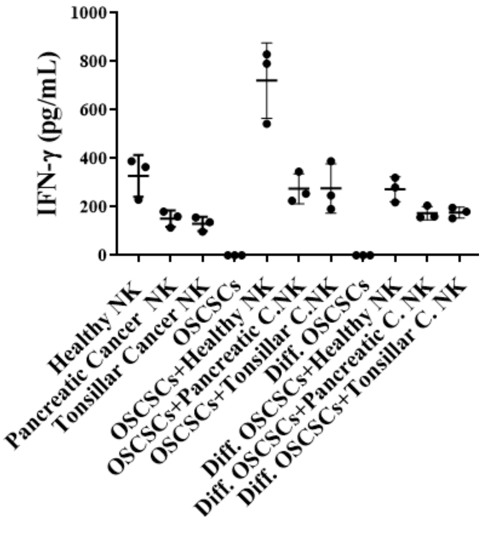

Figure S2. Cancer patients' NK cells secreted low IFN-γ secretions when compared to healthy individuals' NK cells. Purified NK cells (1 × 10^6 cells/ml) of healthy individuals and cancer patients were treated with IL-2 (1000 U/ml) for 18 hours and were co-cultured with OSCSCs and NK-differentiated OSCSCs at tumors: NK ratio of 1:1. On days 1 and 3 after the co-culture, supernatants

Citation: Kaur, K.; Safaie, T.; Ko, M.-W.; Wang, Y.; Jewett, A. ADCC against MICA/B is Mediated against Differentiated Oral and Pancreatic and Not Stem-Like/Poorly Differentiated Tumors by the NK Cells; Loss in Cancer Patients due to Downmodulation of CD16 Receptor. *Cancers* **2021**, *13*, x. https://doi.org/10.3390/xxxxx

Received: 10 December 2020
Accepted: 5 January 2021
Published: date

Publisher's Note: MDPI stays neutral with regard to jurisdictional claims in published maps and institutional affiliations.

Copyright: © 2021 by the authors. Submitted for possible open access publication under the terms and conditions of the Creative Commons Attribution (CC BY) license (http://creativecommons.org/licenses/by/4.0/).

were harvested and IFN-γ secretions were determined using single ELISA (n = 3).

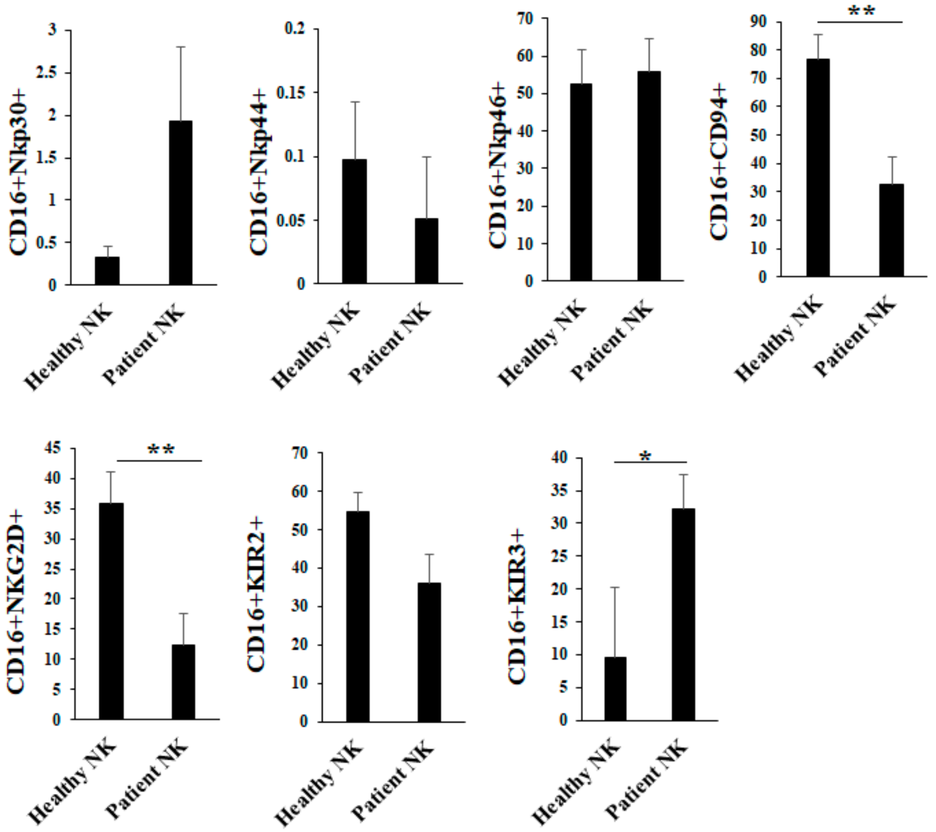

Figure S3. NK cells' surface expression on healthy individual and cancer patients. Freshly purified NK cells from healthy individuals and cancer patients were analyzed for the surface expressions of CD16, Nkp30, Nkp44, Nkp46, CD94, NKG2D, KIR2 and KIR3 using flow cytometry. IgG2 isotype antibodies were used as controls. Average of 8 experiments is shown in Figure. ** (p value 0.001–0.01), * (p value 0.01–0.05)

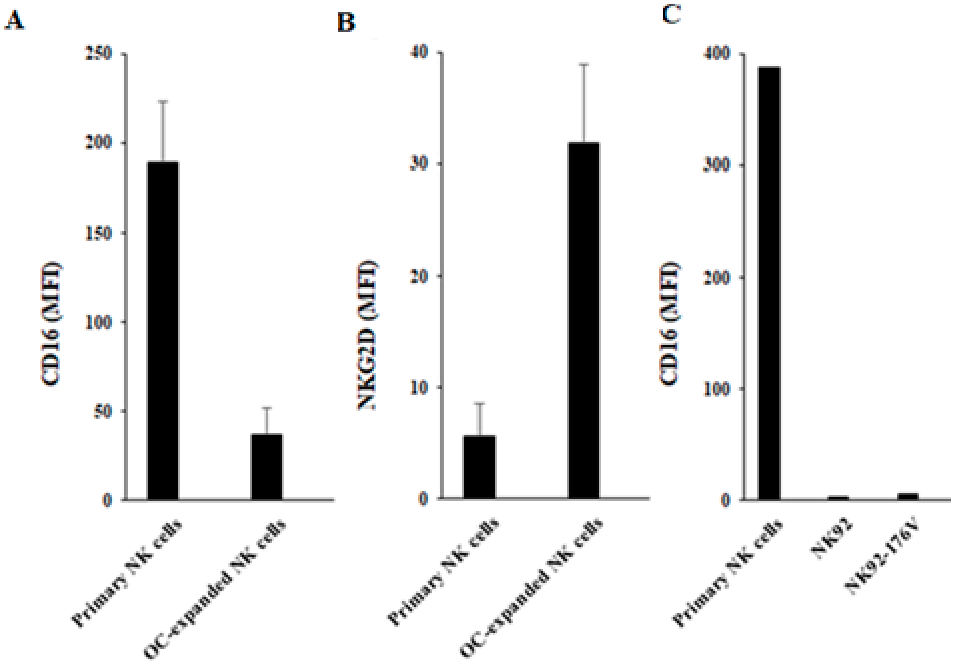

Figure S4. Surface expression levels of CD16 on primary NK cells, OC-expanded NK cells and NK92 cell lines, and NKG2D surface expression level on primary NK and OC-expanded NK cells. OCs were generated as described in the Materials and Methods. NK cells (1×10^6 cells/ml) from healthy individuals were treated with a combination of IL-2 (1000 U/ml) and anti-CD16 mAbs (3 µg/ml) for 18 hours before they were co-cultured with the OCs and sAJ2 at a ratio of 1:2:4 (OCs:NK:sAJ2). Freshly purified primary and OC-expanded NK cells were analyzed for the surface expressions of CD16

(**A**), and NKG2D (**B**) using flow cytometry. IgG2 isotype antibodies were used as controls. Average of 3 experiments is shown in Figures A and B. Freshly purified primary NK cells, NK92 and NK92-176V cells were analyzed for the surface expressions of CD16 using flow cytometry. IgG2 isotype antibodies were used as controls **(C)**. One of the three representative experiments is shown in Figure.

A

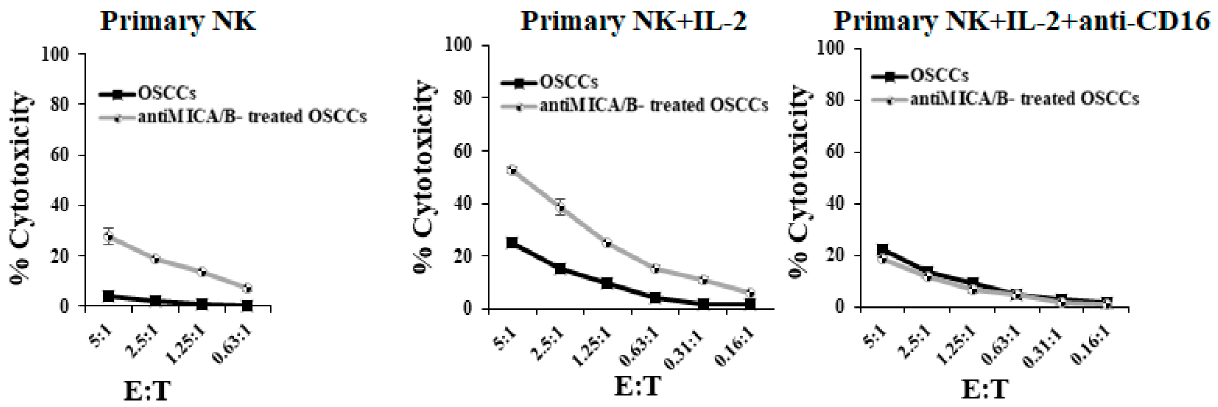

B

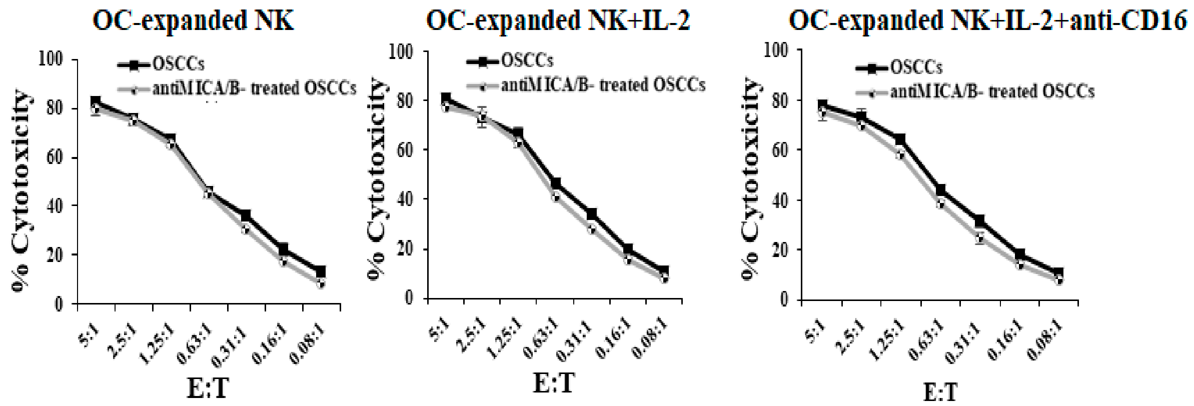

C

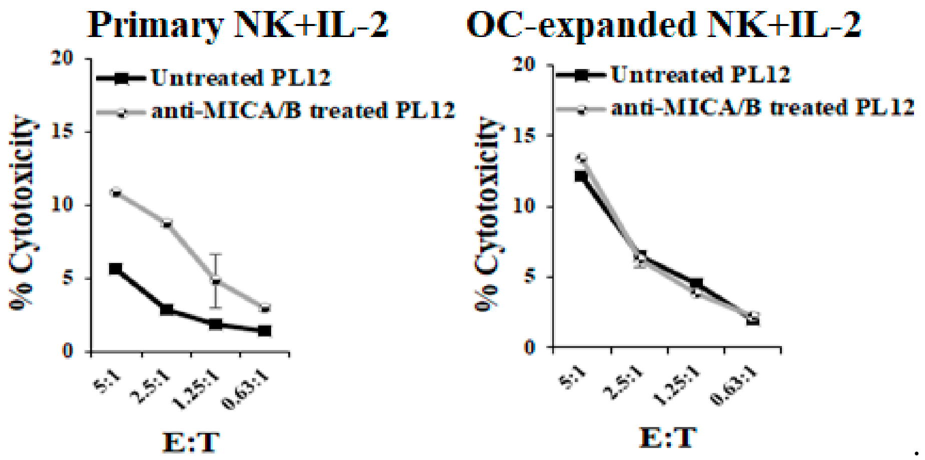

Figure 5. Higher levels of NK cell-mediated ADCC was seen in freshly isolated primary NK cells vs. OC-expanded NK cells. Freshly purified NK cells from healthy individuals were left untreated, treated with IL-2 (1000 U/mL) or a combination of IL-2 and anti-CD16 mAbs (3 µg/ml) for 18 hours and were used as effectors. OSCCs were labeled with ^{51}Cr for an hour after which cells were washed to remove unbound ^{51}Cr. ^{51}Cr-labeled OSCCs were then left untreated or treated with anti-MICA/B mAbs (5 µg/ml) for 30 minutes. The unbounded antibodies were washed and the cytotoxicity against the OSCCs was determined using the standard 4-hour ^{51}Cr release assay. The Lytic units (LU) 30/10^6 cells were determined using the inverse number of NK cells required to lyse 30% of target cells x 100 **(A)**. OCs were generated as described in

Materials and Methods. NK cells (1x10^6 cells/ml) from healthy individuals were treated with a combination of IL-2 (1000 U/ml) and anti-CD16 mAbs (3 µg/ml) for 18 hours before they were co-cultured with the OCs and sAJ2 at a ratio of 1:2:4 (OCs:NK:sAJ2). On day 15, OC-expanded NK cells were washed and left untreated, treated with IL-2 (1000 U/ml), or a combination of IL-2 and anti-CD16 mAbs (3 µg/ml) for 18 hours. OSCCs were labeled with ^{51}Cr for an hour after which cells were washed to remove unbound ^{51}Cr. ^{51}Cr-labeled OSCCs were then left untreated or treated with anti-MICA/B mAbs (5 µg/ml) for 30 minutes. The unbounded antibodies were washed and the cytotoxicity against the OSCCs was determined using the standard 4-hour ^{51}Cr release assay. LU 30/10^6 cells were determined as described in Fig. S5A **(B)**. OCs were generated as described in Materials and Methods. NK cells were prepared and co-cultured with OCs as described in Fig. S5B. On day 15, OC-expanded NK cells were washed and treated with IL-2 (1000 U/ml) for 18 hours. Primary NK cells were also treated with IL-2 (1000 U/ml) for 18 hours. PL12 were labeled with ^{51}Cr for an hour after which cells were washed to remove unbound ^{51}Cr. ^{51}Cr-labeled PL12 were then left untreated or treated with anti-MICA/B mAbs (5 µg/ml) for 30 minutes. The unbounded antibodies were washed and the cytotoxicity against the PL12 was determined using the standard 4-hour ^{51}Cr release assay. LU 30/10^6 cells were determined as described in Fig. S5A **(C)**.

A

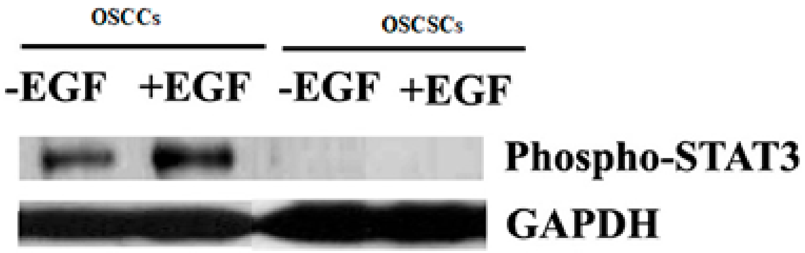

B

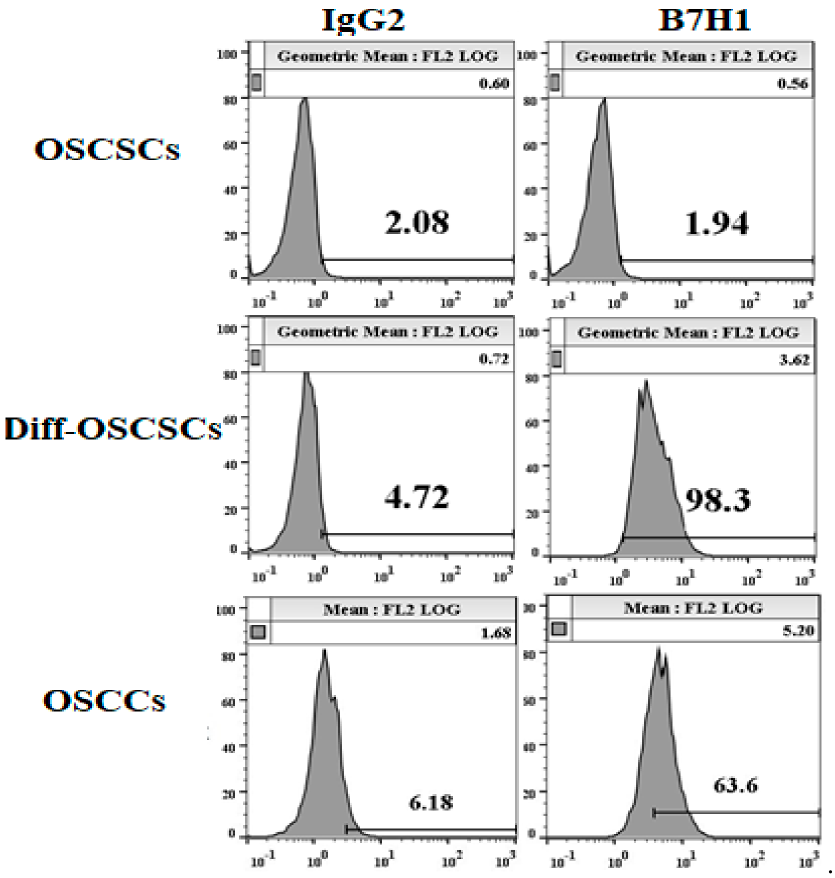

C

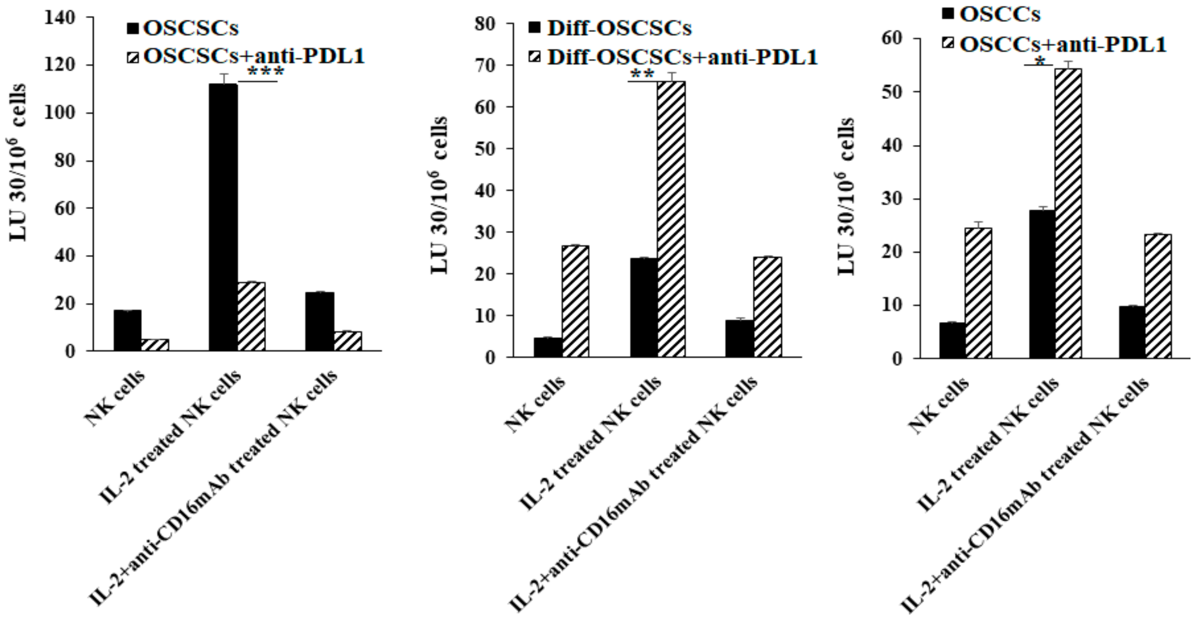
OSCSCs
OSCSCs+anti-PDL1
***
LU 30/10^6 cells
NK cells
IL-2 treated NK cells
IL-2+anti-CD16mAb treated NK cells
Diff-OSCSCs
Diff-OSCSCs+anti-PDL1
**
OSCCs
OSCCs+anti-PDL1
*

D

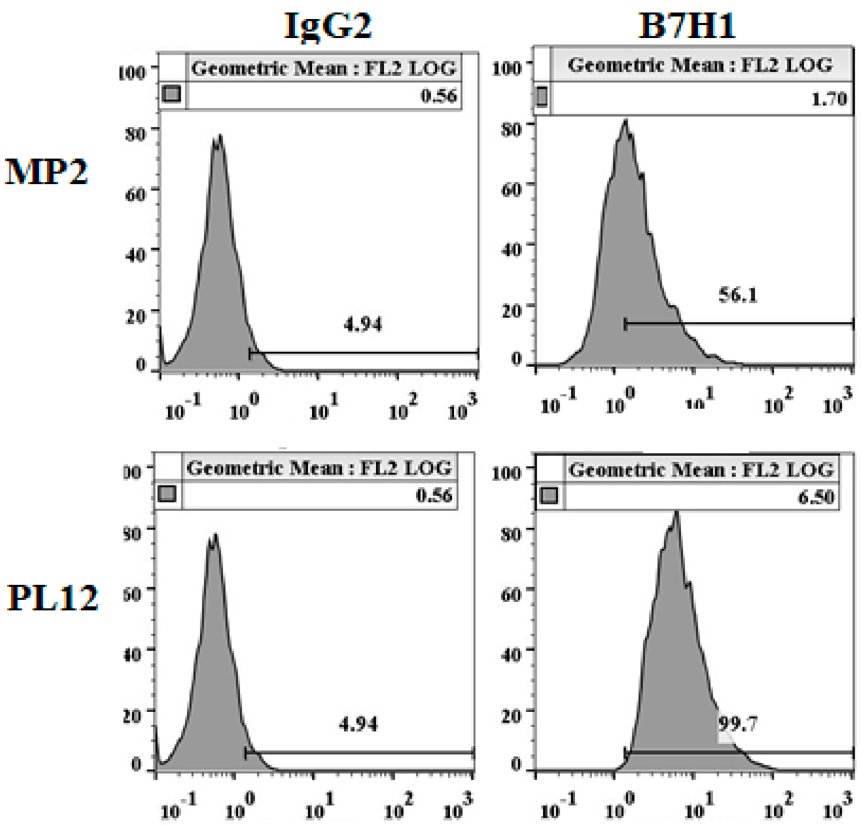
IgG2
B7H1
MP2
PL12
Geometric Mean : FL2 LOG
0.56
1.70
6.50
4.94
56.1
99.7

E

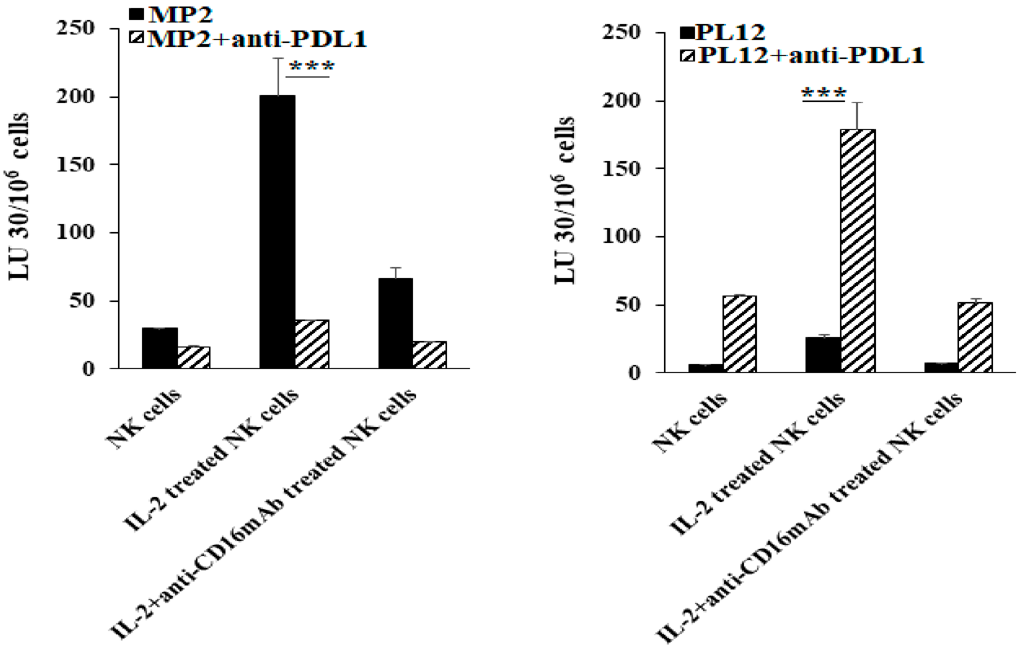

Figure S6. Higher surface expression of EGF and B7H1, and NK cell-mediated ADCC in differentiated tumors in comparison to their stem-like counterparts. The levels of EGF in OSCCs and OSCSCs were determined using Western blot (**A**). The levels of B7H1 expression were determined on OSCSCs, NK-differentiated OSCSCs and OSCCs using surface staining with anti-B7H1 antibodies (**B**). Purified NK cells (1×10^6 cells/ml) were left untreated, treated with IL-2 (1000 U/ml), or treated with IL-2 (1000 U/ml) and anti-CD16 mAbs (3 µg/ml) for 18 hours and used against untreated and anti-PDL1 (20 µg/ml) treated OSCSCs, diff-OSCSCs, and OSCCs in a 4-hour ^{51}Cr release assay. The lytic units (LU) 30/10^6 cells were determined using the inverse number of NK cells required to lyse 30% of target cells × 100 (**C**). The levels of B7H1 expression were determined on MP2 and PL12 tumors using surface staining with anti-B7H1 antibodies (**D**). Purified NK cells (1×10^6 cells/ml) were left untreated, treated with IL-2 (1000 U/ml), or treated with IL-2 (1000 U/ml) and anti-CD16 mAbs (3 µg/ml) for 18 hours and used against untreated and anti-PDL1 (20 µg/ml) treated MP2 and PL12 in a 4-hour ^{51}Cr release assay. The lytic units (LU) 30/10^6 cells were determined using the inverse number of NK cells required to lyse 30% of target cells × 100 (**E**). *** (p value < 0.001), ** (p value 0.001–0.01), * (p value 0.01–0.05)
